# FRDD-Net: Automated Carotid Plaque Ultrasound Images Segmentation Using Feature Remapping and Dense Decoding

**DOI:** 10.3390/s22030887

**Published:** 2022-01-24

**Authors:** Yanhan Li, Lian Zou, Li Xiong, Fen Yu, Hao Jiang, Cien Fan, Mofan Cheng, Qi Li

**Affiliations:** 1 Electronic Information School, Wuhan University, Wuhan 430072, China; liyanhan@whu.edu.cn (Y.L.); zoulian@whu.edu.cn (L.Z.); jh@whu.edu.cn (H.J.); fce@whu.edu.cn (C.F.); chengmofan@whu.edu.cn (M.C.); liqicarology@whu.edu.cn (Q.L.); 2 Cardiovascular Ultrasound Department, Zhongnan Hospital of Wuhan University, Wuhan 430071, China; 3 Department of Ultrasound, Central Hospital of Wuhan, Tongji Medical College, Huazhong University of Science and Technology, Wuhan 430014, China; yufen@zxhospital.com

**Keywords:** ultrasound, segmentation, deep convolutional neural networks, carotid plaques, encoder–decoder

## Abstract

Automated segmentation and evaluation of carotid plaques ultrasound images is of great significance for the diagnosis and early intervention of high-risk groups of cardiovascular and cerebrovascular diseases. However, it remains challenging to develop such solutions due to the relatively low quality of ultrasound images and heterogenous characteristics of carotid plaques. To address those problems, in this paper, we propose a novel deep convolutional neural network, FRDD-Net, with an encoder–decoder architecture to automatically segment carotid plaques. We propose the feature remapping modules (FRMs) and incorporate them into the encoding and decoding blocks to ameliorate the reliability of acquired features. We also propose a new dense decoding mechanism as part of the decoder, thus promoting the utilization efficiency of encoded features. Additionally, we construct a compound loss function to train our network to further enhance its robustness in the face of numerous cases. We train and test our network in multiple carotid plaque ultrasound datasets and our method yields the best performance compared to other state-of-the-art methods. Further ablation studies consistently show the advancement of our proposed architecture.

## 1. Introduction

Atherosclerotic plaques in the internal carotid artery (ICA) is the major cause of cardiovascular diseases, thus causing a high mortality and morbidity globally [1,2]. Research studies [3] show that carotid plaques are considered as valid indicators of atherosclerosis. There are several medical imaging modalities used for carotid plaques, such as computed tomography (CT), magnetic resonance imaging (MRI), X-ray, and ultrasonography (US). Among them, ultrasonography is preferred for its noninvasiveness, ease of operation, affordability, lack of radiation, and portability [4,5,6]. The captured carotid artery ultrasound images provide various information, such as carotid intima-media thickness, plaque location and size, plaque echo intensity, plaque surface morphology, etc. The image information shows the pathological condition and the state of the cardiovascular and cerebrovascular vessels. Therefore, accurate segmentation of carotid plaques is essential for subsequent diagnosis, evaluation, and prognosis. Nevertheless, ultrasound images are of relatively low quality due to echo artifacts and speckle noise; carotid plaques often stick to the blood vessel boundary and the types of plaques are complex, which brings difficulties for manual segmentation. Furthermore, the precision of segmentation results mainly relies on the subjective judgment of sonographers. However, there is a usual shortage of professional and experienced sonographers.

Therefore, research studies about automated carotid plaque segmentation have been widely carried out. Many computer-aided methods of carotid plaque segmentation have been proposed to assist sonographers [7,8,9,10,11]. Carl et al. [12] proposed to automatically delineate the lumen-intima and media-adventitia layer. Zhou et al. [13] proposed to improve basic network structure for the segmentation of carotid lumen-intima boundaries. Those methods mostly adopted deep neural networks (DNNs) [14] such as fully convolution networks (FCNs) [15] and U-Nets [16] to implement segmentation tasks. Such networks can alleviate shortcomings of manual methods. However, there are still several challenges for existing computer-aided methods of carotid plaque ultrasound images segmentation. (1) Components in those networks [12,13,17,18] treat every single value in feature maps equally important, which is often contrary to actual situations. Note that the areas of plaques should be emphasized more. (2) Decoders in mainstream methods [11,16,17] receive features straightly from encoders or through simple skip connections, thus leaving out meritorious intermediate features and causing low-effectiveness fusions. (3) The size of carotid plaques varies widely and those of small size bring more difficulties for the segmentation [19].

In this paper, we present a new approach that deploys a convolution network with an encoder–decoder architecture to automatically segment carotid plaques on ultrasound images, namely FRDD-Net. Specifically, in FRDD-Net, feature remapping modules (FRMs) are proposed and multiple FRMs constitute the encoding and decoding blocks to better extract and process previous features. In the decoder, a dense decoding mechanism is proposed within all the decoding blocks. The dense decoding mechanism exploits multilevel features and their fusions from the encoder step by step, thus elevating the utilization efficiency of features. Additionally, a compound loss function is constructed to facilitate FRDD-Net’s robustness to segment carotid plaques of various sizes. To sum up, the main contributions of our FRDD-Net are as follows:

(1) To mitigate challenge 1, a novel feature remapping module is proposed. FRMs embedded in encoding and decoding blocks can reweight input features to facilitate their rationality.
(2) To mitigate challenge 2, a novel dense decoding mechanism is proposed. Such decoding architecture can exploit hierarchical features along with their fusions to promote segmentation performance.
(3) To mitigate challenge 3, a novel compound loss function is constructed. The loss function can improve FRDD-Net’s reliability when handling intractable cases.

## 2. Related Works

### 2.1. Traditional Methods for the Carotid Ultrasound Image Segmentation

In general, carotid ultrasound images segmentation involves the combination of several components, including ultrasound image preprocessing, feature extraction, and segmentation of the plaques. Most traditional algorithms focused on extracting more representative features from the ultrasound image. Some of them only focused on segmenting vessel boundary. Sumathi et al. [20] attempted to segmentation the intima-media thickness (IMT) of the far wall, using a level set segmentation method based on edge map without reinitialization. They extracted geometric features such as equivalent diameter, solidity, and extent. Zeynettin et al. [9] attempted to segment carotid plaques on B-mode ultrasound (BMUS) and contrast-enhanced ultrasound (CEUS) images simultaneously. Their method consisted of nonrigid motion estimation and compensation, vessel detection, lumen–intima segmentation, and media–adventitia segmentation. Similarly, Diego et al. [21] adopted a nonrigid motion estimation (NME) to improve the signal-to-noise ratio of simultaneously acquired BMUS and CEUS image sequences. Then, an intensity joint-histogram classification and a graph-based segmentation were used to segment the lumen. Other methods focused on segmenting the vessel boundary and plaque. Loizou et al. [7] employed speckle reduction filtering (with the hybrid median filter) and parametric active contours. Francois et al. [8] estimated the motion field and integrated the result into the prior of a Bayesian segmentation model. Christos et al. [10] proposed an integrated system for the segmentation of atherosclerotic carotid plaque in ultrasound images of the common carotid artery (CCA) based on video frame normalization, speckle reduction filtering, M-mode state-based identification, parametric active contours, and snake segmentation.

The main purpose of these traditional methods was to design or extract more representative manual features from carotid artery ultrasound images. Although substantial progress has been made in the field of vessel boundary and plaque segmentation, traditional algorithms still have shortcomings that cannot be ignored. Methods based on the geometrical, grayscale, and texture features of ultrasound images have poor robustness due to the low quality of ultrasonic imaging. Furthermore, manually selected features are subjective, which may lack representativeness. The result is that the segmentation is not accurate enough and lacks robustness.

### 2.2. Deep Neural Networks for the Segmentation of Carotid Plaque Ultrasound Image

The segmentation needs to exactly match the vessel boundary and plaque at the pixel level, which requires methods to have outstanding feature extraction capabilities. Profiting from the development of deep learning (DL) [14], deep neural networks (DNNs), particularly those involving convolutional neural networks (CNNs), can effectively extract abstract features of high dimensions from ultrasound images. Menchon-Lara et al. [17] used standard multilayer perceptrons (MLPs) with one single hidden layer, trained under the scaled conjugate gradient (SCG) rule to carry out the segmentation of CCA ultrasound images. Besides, CNNs take into account the spatial distribution of input images. Furthermore, the output feature maps retain the spatial information of the object. Shin JY et al. [18] presented a unified framework based on a CNN with a LeNet-like architecture to automate and accelerate carotid intima-media thickness CIMT video interpretation. Long J et al. [15] proposed fully convolution network (FCN) for segmentation. The FCN contains no fully connected layer to adapt to variable input sizes. Furthermore, the deconvolutional layer that outputs fine results allows the network to handle segmentation tasks. Ran et al. [22] proposed a voxel-based fully convolution network (Voxel-FCN) and a continuous max-flow module to conduct automated segmentation tasks. For networks with an encoder–decoder architecture, U-Net [16] has been widely applied to the medical segmentation field. Its encoder extracts high-level semantic information gradually and the decoder restores the original resolution. Carl et al. [12] used a simplified U-Net for delineating both the lumen-intima layer and the media-adventitia layer. They developed a new geometrically constrained objective function as part of the network’s stochastic gradient descent optimization. Azzopardi et al. [23] proposed to use DNNs with an encoder–decoder structure as a segmentation tool and evaluated the effects of its hyperparameters on segmentation performance. Zhou et al. [13] used a dynamic CNN model to fit carotid images of different subjects for the segmentation of media-adventitia boundaries and improved U-Net network structure for the segmentation of lumen-intima boundaries. Meiyan et al. [11] modified U-Net models and used an ensemble of separate decoders for vessels and plaques segmentation tasks. Perez et al. [24] introduced a general condition layer named feature-wise linear modulation to handle original features through affine transformation. Similarly, Hu et al. [25] introduced a squeeze-and-excitation mechanism to modulate features by their channels, generating more rational representations.

Although these methods have achieved substantial success in the segmentation of carotid plaque ultrasound images, there are still some limitations. On the one hand, it remains a challenging task for DNNs to extract features from ultrasound images of low contrast and quality. Moreover, carotid plaques are usually of irregular shapes and diverse sizes. One the other hand, large pixel-level annotated datasets are required to develop effective and feasible segmentation methods. However, the current datasets cannot meet such requirements.

## 3. Materials and Methods

### 3.1. Data Preprocessing

Due to the limited amount of training data in our dataset, we used data augmentation techniques for image processing. Data augmentation strategies have been proven to help prevent network from overfitting and promote a network’s generalization ability. Data augmentation can be through random image geometric transformations, including rotation, scaling, flipping, and movement, artificially increasing the training image data. In addition, it can ensure that the model used focuses on carotid plaque and not various noise sources. All enhanced images were resized to 256×256 pixels for standardization.

### 3.2. Overall Architecture

**Figure 1:**
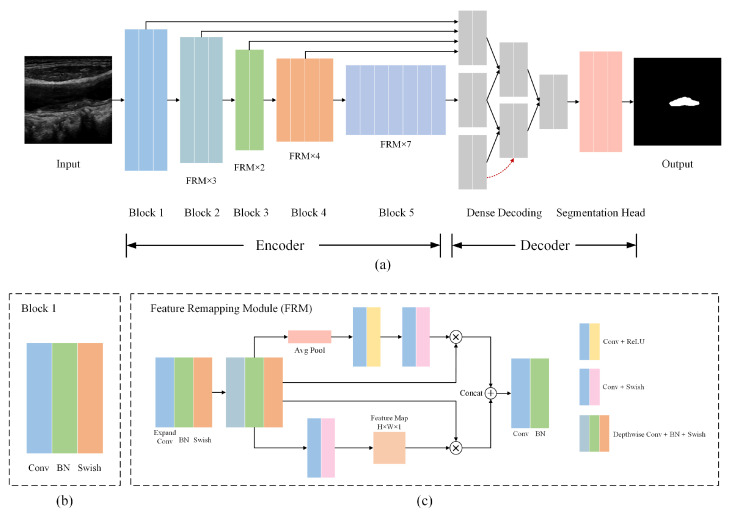
Overall architecture of FRDD-Net. (**a**) Full flowchart of FRDD-Net. (**b**) Detailed structure of encoding block 1. (**c**) Detailed structure of feature remapping modules (FRMs).

The detailed architecture of the proposed FRDD-Net is shown in Figure 1a. The designed network has a novel encoder–decoder architecture. The encoder contains a series of encoding blocks embedded with FRMs and can generate feature maps of different levels as plural inputs of the decoder. Similarly, the decoder also contains a series of decoding blocks embedded with FRMs. Moreover, the dense decoding mechanism in the decoder employs multilevel features with their fusion to acquire segmentation results. In practice, ultrasound images are first resized to 256×256 pixels as the input of the encoder. Then, each encoding block of the encoder extracts its own feature map and 5 feature maps from low level to high level are obtained. The dense decoding mechanism utilizes those 5 feature maps along with their specific concatenations step by step, producing hierarchical decoded features. The feature from the last decoding block is used by the segmentation head to acquire the final results. The detailed architectures of the decoder and encoder are discussed in the following part.

### 3.3. Feature Remapping Module

Notably, ultrasound images of carotid plaques contain substantial redundancy, namely, tissues irrelevant to nidi. Previous works treat the extracted features as equally crucial, which may lead to misleading results. To tackle this problem, we propose FRMs to differentiate the spatial-wise and channel-wise contributions of the original feature maps. It can help the network to focus more on the correlative information of carotid plaques and alleviate the flaw mentioned in challenge 1.

As shown in Figure 1a, the encoder of FRDD-Net is composed of 5 encoding blocks. Except for the first block, the other 4 encoding blocks have similar structures. The detailed structure of the first encoding block is presented in Figure 1b. It consists of a 3×3×2 convolution (Conv) layer, a batch normalization (BatchNorm) layer, and a swish layer. Furthermore, the other 4 encoding blocks are all composed of multiple FRMs with different sizes.

In the FRM, as shown in Figure 1c, the input feature map *F* is processed by two branches concurrently. In the upper branch, the input feature map *F* is firstly processed by depthwise convolution [26] and batch normalization. Furthermore, the acquired feature map $F^{\prime }$ with size of H×W×C is further processed by global average pooling to build a new global channel feature $G_{c}$ with size of 1×1×C, where $G_{c}=\frac{1}{H\times W}\sum_{h}\sum_{w}F^{\prime }$. To obtain the remapping features, an attention mechanism [27] is exploited in this module. For channel-wise remapping, a reducing convolution layer, a swish layer, an expanding layer, and a sigmoid layer are employed on $G_{c}$ so as to build the remapping parameters of channel $G_{c}^{\prime }$. Subsequently, elements in $G_{c}^{\prime }$ and *F* are multiplied to obtain the channel remapping feature $F_{c}$. In total, $F_{c}$ can be expressed as follows:

(1) $$\left\{\begin{array}{cc} F^{\prime }=\Phi_{bn}\left(\Phi_{dc}\left(F\right)\right) & \\ G_{c}=\Phi_{avgp}\left(F^{\prime }\right) & \\ G_{c}^{\prime }=\sigma \left(\Phi_{ec}\left(\varepsilon \left(\Phi_{rc}\left(G_{c}\right)\right)\right)\right) & \\ F_{c}=F⊙G_{c}^{\prime } \end{array}\right.$$

where $\Phi_{dc}$ is a depthwise convolution, $\Phi_{bn}$ is a batch normalization, $\Phi_{avgp}$ is a global average pooling, $\Phi_{rc}$ is a reducing convolution, ε is a swish function, $\Phi_{ec}$ is an expanding convolution, σ is a sigmoid function, and ⊙ is an element-wise product.

In the lower branch, a similar spatial-wise remapping procedure is conducted. Analogously, *F* is processed by a reducing convolution and a sigmoid function to obtain the global spatial feature map $G_{s}$ with a size of H×W×1. Then, a channel-wise average pooling is applied to $G_{s}$ to generate the pooled feature map $G_{sa}$. Subsequently, a convolution layer and a sigmoid layer are applied to $G_{sa}$ to obtain the remapping parameters of spatiality $G_{s}^{\prime }$. Subsequently, elements in $G_{s}^{\prime }$ and *F* are multiplied to obtain the spatial remapped feature $F_{s}$. In summary, $F_{s}$ can be expressed as follows:

(2) $$\left\{\begin{array}{cc} G_{s}=\sigma \left(\Phi_{rc}\left(F\right)\right) & \\ G_{sa}=\Phi_{cap}\left(G_{s}\right) & \\ G_{s}^{\prime }=\sigma \left(\Phi_{c}\left(G_{sa}\right)\right) & \\ F_{s}=F⊙G_{s}^{\prime } \end{array}\right.$$

where $\Phi_{cap}$ is the channel-wise average pooling.

After acquiring the channel-wise remapping $F_{c}$ and the spatial-wise remapping $F_{s}$, those two remapped features are concatenated to form the final remapping $F_{rm}$. At last, $F_{rm}$ is convolved to the desired dimension as the output $F_{o}$. Formally, $F_{o}$ is expressed as follows:

(3) $$\left\{\begin{array}{cc} F_{rm}=F_{c}⊕F_{s} & \\ F_{o}=\Phi \left(F_{rm}\right) & \end{array}\right.$$

where ⊕ is the concatenation operation and Φ denotes the convolution operation.

Figure 1a demonstrates that the second, third, fourth, and fifth encoding blocks have 3, 2, 4, and 7 FRMs, respectively. The 5 encoding blocks generate 5 feature maps of different levels and all feature maps are densely decoded by the proposed decoder. The details of the proposed decoder is discussed in the following part.

### 3.4. Dense Decoding Mechanism

As mentioned before, the encoder of FRDD-Net generates 5 feature maps of different levels. Judicious utilization of multilevel features can considerably ameliorate segmentation performance. Unet++ [28] is a widely used architecture in medical image segmentation and its nested decoding mechanism exploits multilevel features to their full extent. We ameliorate such strategy and embed FRMs in decoding blocks to construct a dense decoding mechanism. The proposed dense decoding mechanism can achieve better productiveness and maintain convincing performance.

**Figure 2:**
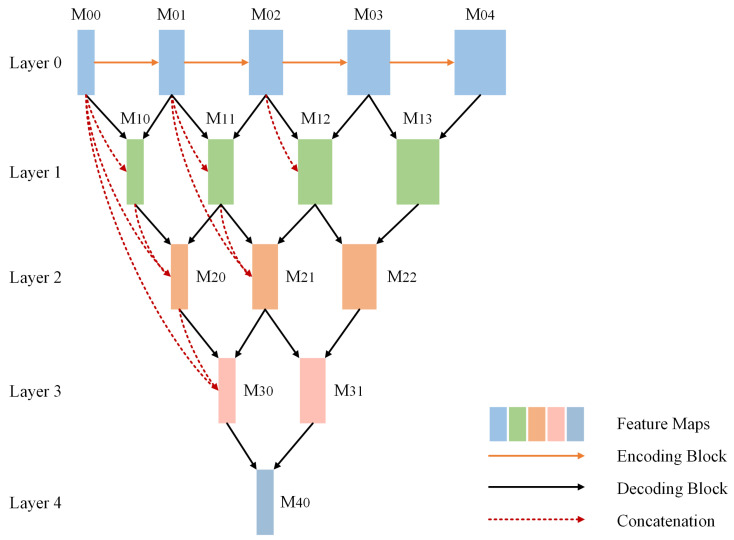
Architecture of dense decoding mechanism in the decoder.

The details of the dense decoding mechanism are presented in Figure 2. The 5 extracted features are densely decoded by similar decoding blocks. On layer 0, $M_{00}$, $M_{01}$, $M_{02}$, $M_{03}$, and $M_{04}$ are feature maps generated by the first, second, third, fourth, and fifth encoding blocks, respectively. Among those 5 feature maps, two adjacent maps, namely, $M_{00}$ and $M_{01}$, $M_{01}$ and $M_{02}$, $M_{02}$ and $M_{03}$, and $M_{03}$ and $M_{04}$ are decoded together by four decoding blocks to form elements on the next layer. Next, on layer 1, between two adjacent elements, the one generated from lower-level features is concatenated with elements from the previous layer to form the fusion feature before being encoded. Namely, to obtain $M_{20}$, $M_{10}$ is first concatenated with $M_{00}$. Then, the fusion feature and $M_{11}$ are decoded together by decoding blocks to form $M_{20}$. Similarly, on layer 2, the concatenation of $M_{20}$, $M_{10}$, $M_{00}$ are decoded together with $M_{20}$ to obtain $M_{30}$. Procedures are exactly the same for layer 3 and layer 4. Formally, the elements in the decoding structure are calculated as follows:

(4) $$M_{i,j}=\left\{\begin{array}{cc} E(M_{i,j-1}) & i=0\\ D({\left[M_{k,j}\right]}_{k=0}^{i-1},M_{i-1,j+1}) & i>0 \end{array}\right.$$

where function E(·) is the encoding block, D(·) is the decoding block, and [·] denotes the concatenation operation. Basically, elements at layer i=0 are the outputs of the previous encoder. Furthermore, elements at layer i>0 are obtained as previously mentioned. Such a dense decoding structure can utilize features from preceding layers well, creating abundant representations, which addresses the problem challenge 2. It is beneficial to apply that mechanism to carotid ultrasound images, which usually have unsatisfactory imaging quality.

**Figure 3:**
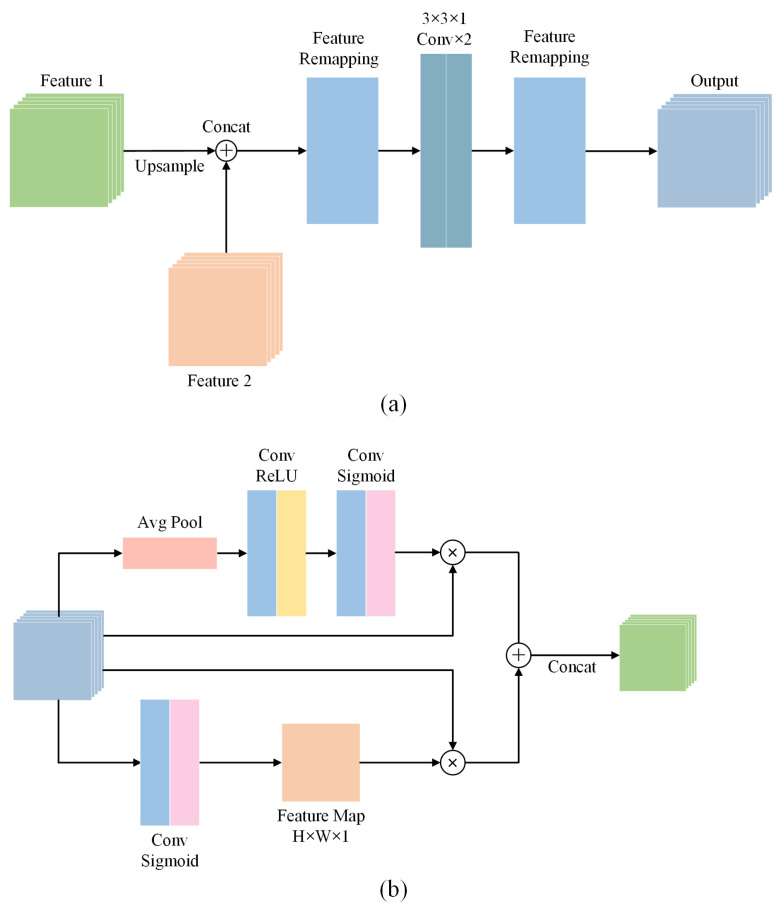
Architecture of decoding blocks. (**a**) Detailed structure of decoding blocks. (**b**) Detailed structure of feature remapping modules in the decoder.

The detailed structure of the decoding blocks are shown in Figure 3a. The two input features from the previous layers are first concatenated, and the concatenated feature is reconstructed by two FRMs. As shown in Figure 3b, the structure of FRM in decoding blocks is similar to that in encoding blocks, except for some convolutional layers at the beginning and the end.

### 3.5. Compound Loss Function

When training FRDD-Net, all carotid ultrasound images along with their masks are resized to 256×256 pixels. As mentioned in challenge 3, the size of carotid plaques varies widely and some of the carotid plaques are relatively small compared to the whole ultrasound image, leading to imbalanced pixel-wise categories and bringing challenges to segmentation tasks. To cope with this problem, we constructed a compound loss function to enhance FRDD-Net’s robustness when encountering such cases. The whole compound loss function was defined as follows:

(5) $$L=\alpha \cdot L_{DL}+\beta \cdot L_{FTL}$$

where $L_{DL}$ is the dice Loss [29], $L_{FTL}$ is the focal Tversky term [30]. α and β are the weights to balance the aforementioned two terms. Dice loss is commonly used in medical image segmentation for its direct optimization on dice similarity coefficients (DSCs). Furthermore, its definition is:

(6) $$L_{DL}=\sum_{C}\left(1-DSC_{C}\right)$$

where $DSC_{c}$ is the DSC for category *C*.

We concentrate on the second term of the compound loss function, the focal Tversky term [30]. The focal Tversky term can alleviate networks’ failure on highly imbalanced data and small region of interests (RoIs). It is defined as follows:

(7) $$FTL_{C}=\sum_{C}{\left(1-TI_{C}\right)}^{\frac{1}{\gamma }}$$

where $TI_{c}$ is the Tversky similarity index [31], and it can be expressed as follows:

(8) $$TI_{C}=\frac{\sum_{N}^{i=1}p_{iC}g_{iC}+\varepsilon }{\sum_{N}^{i=1}p_{iC}g_{iC}+\lambda \sum_{N}^{i=1}p_{i\bar{C}}g_{iC}+\sigma \sum_{N}^{i=1}p_{iC}g_{i\bar{C}}+\varepsilon }$$

where $p_{iC}$ is the probability that pixel *i* belongs to the lesion class *C* and $p_{i\bar{C}}$ is the probability pixel *i* belongs to the nonlesion class $\bar{C}$. $g_{iC}$ is the ground truth label that pixel *i* belongs to the lesion class *C* and $g_{i\bar{C}}$ is the ground truth label that pixel *i* belongs to the nonlesion class $\bar{C}$. *N* is the total number of pixels in a single image. ε is to prevent division by zero. Hyperparameters λ and σ are to shift the emphasis to improve recall in the case of large class imbalance. γ varies in the range from 1 to 3 to adjust the network’s concentrations on small RoIs.

## 4. Results and Discussions

### 4.1. Dataset and Implementation Details

The ultrasound images used in the experiments were provided by the Department of Ultrasound, Zhongnan Hospital of Wuhan University. The ultrasound images used in the experiments were collected by a GEE95 ultrasonographic equipment. The probe was a 9L linear array probe, the center frequency was 9 MHz, the scanning speed was 3 mm/s, and the scanning distance was about 4 cm. Images were saved in .jpg format. A total of 4384 ultrasound images were obtained. Annotations for the carotid plaques were performed by experienced sonographers on the original ultrasound images. Then, the original ultrasound images along with their masks were preprocessed according to an input size of 256×256 pixels.

A set of 3681 images was selected as the training set and a set of 411 images was selected as the validation set. The rest were selected as the test set. When training and testing the network, the test time augmentation (TTA) mechanism was adopted for the procedure. TTA creates multiple augmented copies of each image in the dataset, having the model make a prediction for each, then returning an ensemble of those predictions to better improve the performance of the model. The augmentation procedure included sharpening, affine transformation, elastic transformation, contrast enhancement, blurring, and coarse dropout.

The proposed FRDD-Net was implemented using Pytorch [32]. The initial learning rate was $1\times 10^{-4}$ and the total number training epochs was 100. During the training procedure, the cosine annealing algorithm with warm up [33] was selected as the decaying scheduler. The number of warmup epochs was five and the learning rate decayed by 0.1 every 10 epochs after epoch 40. Adam [34] with default parameters was adopted as the optimizer. The hyperparameters of FRDD-Net were set as: λ=0.3, σ=0.7, α=0.5, β=0.5, and γ=1.5. Additionally, a 10-fold cross-validation method was adopted during the training and validation procedure to reinforce the reliability and generalization capacity of our model.

### 4.2. Qualitative and Quantitative Analysis of Carotid Plaque Segmentation

In this section, we present the qualitative and quantitative analyses of the segmentation results of carotid plaques. To validate the effectiveness of our proposed model, we compared the performance of FRDD-Net with that of Unet [16], Unet++ [28], DeepLabV3 [35], DeepLabV3+ [36], and PSPNet [37]. All comparative methods were trained and tested with the same strategy as FRDD-Net’s. The initial learning rate was $1\times 10^{-4}$ and the total number of training epochs was 100. The hyperparameters of those methods were set as: λ=0.3, σ=0.7, α=0.5, β=0.5, and γ=1.5.

**Figure 4:**
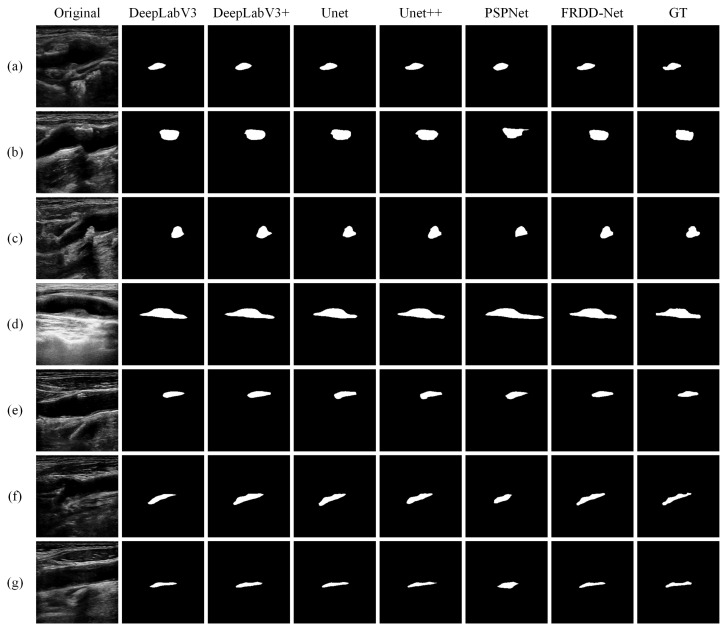
Qualitative comparison of carotid plaque segmentation results produced by FRDD-Net and other methods (DeepLabeV3, DeepLabeV3+, Unet, Unet++, and PSPNet) against ground truth (GT). (**a**–**g**) are partial segmentation results.

The qualitative visual comparisons of segmentation results of the carotid plaques using our proposed method and other state-of-the-art methods are shown in Figure 4. We can see that FRDD-Net outperforms all the other mainstream methods. Due to its poor quality, the carotid plaque on an ultrasound image is liable to be confused with surrounding tissues, leading to dissatisfactory segmentation. The examples are Figure 4b, the 6th image of Figure 4d, the 4th, 5th, and 6th images of Figure 4e. Those methods regard surroundings as targets, producing overlarge segmentation, while FRDD-Net ably alleviate such failure. Another typical example is that some methods fail to correctly segment the edge of targeted carotid plaques (the 3rd and 6th images of Figure 4a, the 2nd, 5th, and 6th images of Figure 4c, the 2nd image of Figure 4f). Those methods are apt to have the segmentation results truncated at the edge of carotid plaques, while FRDD-Net produces comparatively smooth and accurate edges. As for carotid plaques of irregular shapes (Figure 4f,g), other methods such as DeepLapV3, Unet++, and PSPnet output undesired results with blurry boundaries, while FRDD-Net generates the most proximate boundaries. Additionally, the size of carotid plaques in our collected dataset varies widely. For instance, there are normal sizes (Figure 4d,f) and small sizes (Figure 4a,c). The results from Figure 4 show that our method performs the best in both normal and small targets. In general, visual comparison results demonstrate that our FRDD-Net presents a credible and robust ability to segment carotid plaque in ultrasound images in various scenarios.

Table 1 summarizes the quantitative comparison of segmentation results of carotid plaques. It can be observed that FRDD-Net consistently outperforms other methods on both DSC and intersection over union (IoU). Specifically, FRDD-Net yielded a DSC of 83.65% and an IoU of 78.18%, with an improvement of 1.26% in DSC and 2.13% in IoU compared to those in U-net (the method in second place). Note that the baseline of all other methods was efficientnet [38], which is an advanced architecture for encoding. Furthermore, our proposed encoder is referred to as FR-encoder in Table 1. As shown in the penultimate row of Table 1, to verify the effectiveness of our FR-encoder, the encoder of FRDD-Net was replaced with efficientnet-b0 and yielded a DSC of 83.20% and an IoU of 77.41%, better than those of other mainstream methods. This indicates that the FR-encoder has comparatively stronger capability to extract features from original inputs. Still, FRDD-Net with the FR-encoder maintains the best performance, proving the superiority of the proposed dense decoding architectures.

### 4.3. Cross-Dataset Studies

**Figure 5:**
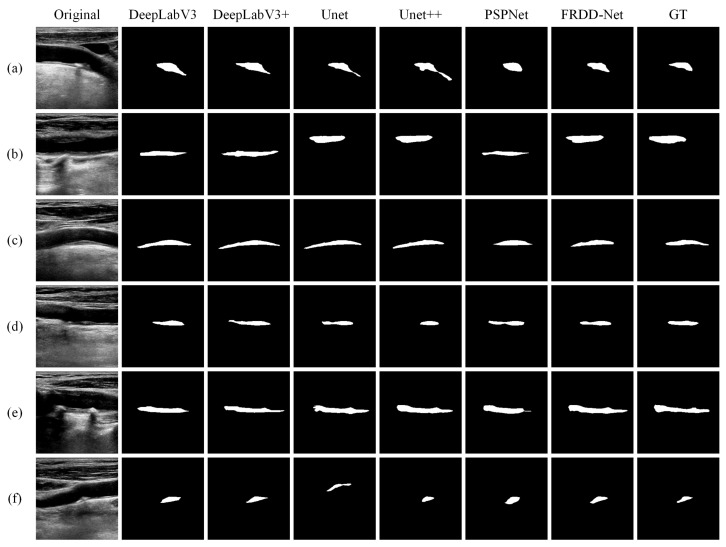
Qualitative comparison of cross-dataset test results produced by FRDD-Net and other methods (DeepLabeV3, DeepLabeV3+, Unet, Unet++, and PSPNet) against ground truth (GT). (**a**–**f**) are partial segmentation results.

To further explore the robustness of FRDD-Net, a cross-dataset experiment was included. Apart from the dataset used for training, validation, and test, an extra set of 431 images was collected to conduct a cross-dataset test. The extra images were acquired from different patients with disparate devices. All aforementioned methods were tested on the extra dataset and the qualitative visual comparisons of segmentation results are presented in Figure 5.

The qualitative results indicate that FRDD-Net maintains the best performance compared to the compared methods. Concretely, in Figure 5a, Unet and Unet++ generated incorrect segmentation. Similarly, in Figure 5b, DeepLabV3, DeepLabV3+, and PSPNet failed to recognize the plaque, whereas FRDD-Net consistently obtained remarkable outcomes. Additionally, in Figure 5c,d, FRDD-Net generated the most accurate results, indicating its strong generalization ability when confronting fire-new cases. Moreover, when encountering intractable cases, for example, plaques with complicated borders (Figure 5e) or small sizes (Figure 5f), other methods either produced blurry boundaries or entirely failed to segment, while FRDD-Net still achieved satisfactory segmentation results. Generally, qualitative results on the cross-dataset test validate that FRDD-Net has a high robustness towards unacquainted scenarios.

In addition, Table 2 presents a quantitative comparison of the cross-dataset test results. On the extra dataset, FRDD-Net yielded a DSC of 82.61% and an IoU of 70.69%, achieving the best performance among all tested methods as well. As mentioned in the qualitative analysis, other methods failed to segment a number of cases, thus causing lower DSC and IoU, while FRDD-Net suffered little from this. Notably, FRDD-Net possessed the smallest gap with the results in internal test among all utilized methods, also indicating its high robustness and generalization ability.

### 4.4. Ablation Studies

**Figure 6:**
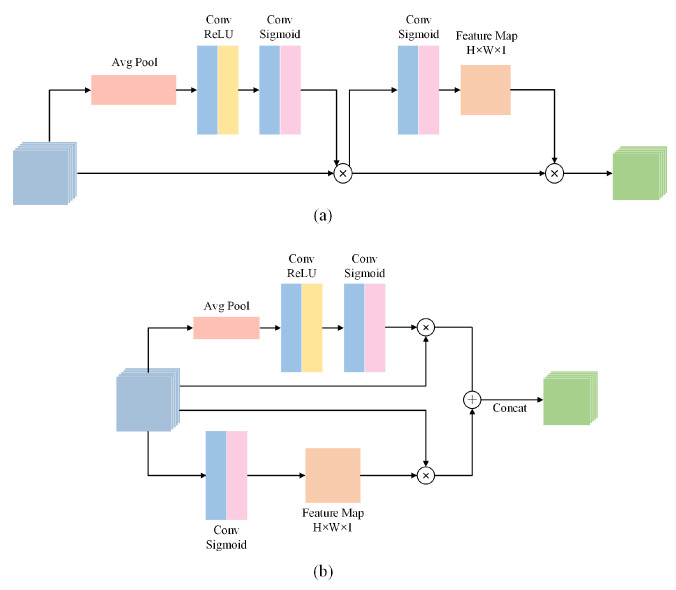
Two forms of feature remapping module. (**a**) Cascaded feature remapping module (C-FRM). (**b**) Parallel feature remapping module (P-FRM).

To further validate the superiority of FRDD-Net, a series of ablation experiments were conducted. Firstly, the proposed FRM is discussed, and we performed the following experiments: removing the FRM from decoding blocks or modifying the structure of FRMs in the decoder and encoder. Except for the aforementioned structure of FRMs, we also tried to employ another structure of FRMs. As shown in Figure 6, we attempted to incorporate two forms of FRMs into FRDD-Net, namely, a cascaded feature remapping module (C-FRM) and a parallel feature remapping module (P-FRM). In C-FRM, the two individual branches were replaced with a cascaded one. Concretely, features passed through spatial remapping and channel remapping sequentially. We combined different FRMs in the encoder and decoder to construct six kinds of varietal FRDD-Net: (a) C-FRMs in the encoder and no FRM in the decoder; (b) P-FRMs in the encoder and no FRM in the decoder; (c) C-FRMs in the encoder and C-FRMs in the decoder; (d) P-FRMs in the encoder and C-FRMs in the decoder; (e) C-FRMs in the encoder and P-FRMs in the decoder; (f) P-FRMs in the encoder and P-FRMs in the decoder. We trained and tested those six varietal networks with the same parameters.

**Figure 7:**
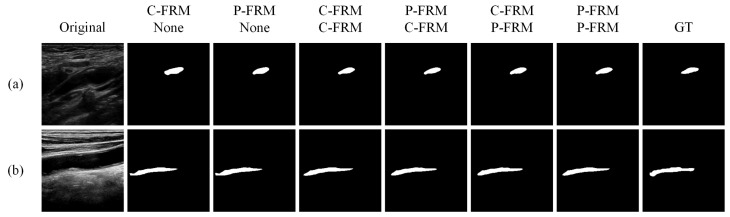
Qualitative comparison of carotid plaque segmentation results produced by different encoding and decoding blocks. (**a**,**b**) are partial segmentation results.

The qualitative results are shown in Figure 7. In Figure 7a, we can see that the network with C-FRMs in the encoder and no FRMs in the decoder fails to segment the contour on the left, while other combinations have comparable performance. In Figure 7b, it is apparent that networks with no FRMs in the decoder perform significantly worse than those with FRMs. Note that the first two networks fail to segment the left edge of the plaque. The quantitative results are shown in Table 3. From the results, we can conclude: (1) networks with FRMs perform better than those without FRMs, indicating the validity of our proposed FRMs; (2) networks with different FRMs perform with no prominent distinctions. Note that the network with P-FRMs in the encoder and P-FRMs in the decoder performs slightly better than other networks especially those with C-FRMs. The reason is that P-FRMs have parallel structures, which can better extract the features from the original input directly in both channel and spatial domains, and features in C-FRMs may degrade due to C-FRMs’ cascaded structures. Therefore, we adopted P-FRMs in our final model.

**Figure 8:**
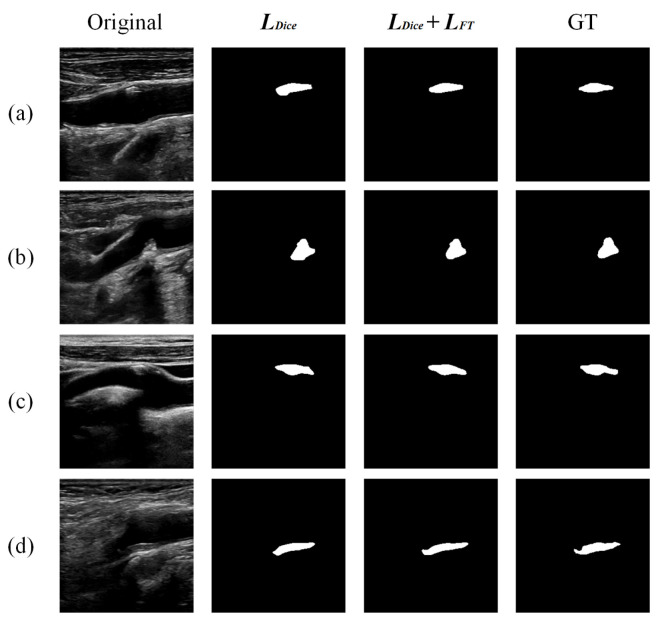
Qualitative comparison of carotid plaque segmentation results produced by different loss functions. (**a**–**d**) are partial segmentation results.

The compound loss function in FRDD-Net was also investigated. We removed the second term, namely, the focal Tversky term from the compound loss to train and test FRDD-Net. The qualitative results are shown in Figure 8. Note that all selected carotid plaques are of relatively small sizes. Figure 8 indicates that a network without focal Tversky term is apt to obtain larger margins, which results in dissatisfactory segmentation. Figure 8a–c) are examples of such cases. The images in Figure 8d are opposite cases and the network without focal Tversky term is unable to segment the complete plaque, having the edge cut off. The quantitative results are shown in Table 4. Those results demonstrate that the compound loss function obviously improves FRDD-Net. Furthermore, it is easy to interpret that since the focal Tversky term of the compound loss is appropriate for data with imbalanced categories and with small RoIs, it fits well with ultrasound images of small carotid plaques.

## 5. Conclusions

In this paper, we present a novel encoder–decoder structure for automated segmentation of carotid plaques in ultrasound images, namely FRDD-Net. In FRDD-Net, we proposed FRMs and embedded them in encoding and decoding blocks to better tackle features from ultrasound images. Moreover, we proposed a dense decoding mechanism in the decoder to handle and ameliorate encoded features to a full extent. Additionally, when training FRDD-Net, we constructed a compound loss function to further elevate its performance regarding intractable cases.

Experimental results demonstrated that FRDD-Net produced a more accurate segmentation of carotid plaque ultrasound images than state-of-the-art methods. A cross-dataset test also indicated that when confronted with unacquainted scenarios, FRDD-Net showed a stronger robustness and generalization ability, which makes FRDD-Net a potential candidate for adoption in a wider range of medical segmentation tasks.

## Figures and Tables

**Table 1:** Overall quantitative comparison results of the carotid plaques in terms of dice similarity coefficients (DSCs) and intersection over union (IoU).

Method	Baseline	DSC (%)	IoU (%)
PSPNet	efficientnet-b0	75.76	65.72
DeepLabV3	efficientnet-b0	82.18	75.68
DeepLabV3+	efficientnet-b0	81.36	74.37
U-net	efficientnet-b0	82.39	76.05
U-net++	efficientnet-b0	82.19	75.71
FRDD-Net	efficientnet-b0	83.20	77.41
**FRDD-Net**	**FR-encoder**	**83.65**	**78.18**

**Table 2:** Overall quantitative comparison results of cross-dataset test in terms of dice similarity coefficients (DSCs) and intersection over union (IoU).

Method	Baseline	DSC (%)	IoU (%)
PSPNet	efficientnet-b0	68.56	55.47
DeepLabV3	efficientnet-b0	71.69	59.41
DeepLabV3+	efficientnet-b0	71.15	59.58
U-net	efficientnet-b0	77.73	66.80
U-net++	efficientnet-b0	80.54	68.24
**FRDD-Net**	**FR-encoder**	**82.61**	**70.69**

**Table 3:** Quantitative comparison results of different encoding and decoding blocks in terms of dice similarity coefficients (DSCs) and intersection over union (IoU).

Encoder	Decoder	DSC (%)	IoU (%)
C-FRM	None	82.23	75.80
P-FRM	None	82.46	76.18
C-FRM	C-FRM	83.26	77.51
P-FRM	C-FRM	83.59	78.06
C-FRM	P-FRM	83.54	78.00
**P-FRM**	**P-FRM**	**83.65**	**78.18**

**Table 4:** Quantitative comparison results of different loss functions in terms of dice similarity coefficients (DSC) and intersection over union (IoU).

Loss Function	DSC (%)	IoU (%)
$L_{Dice}$	82.29	75.88
** $L_{Dice}+L_{FT}$ **	**83.65**	**78.18**

## Data Availability

The data presented in this study are available on request from the corresponding author. The data are not publicly available due to privacy.
